# IL-15 Activates the Jak3/STAT3 Signaling Pathway to Mediate Glucose Uptake in Skeletal Muscle Cells

**DOI:** 10.3389/fphys.2016.00626

**Published:** 2016-12-20

**Authors:** James E. Krolopp, Shantaé M. Thornton, Marcia J. Abbott

**Affiliations:** ^1^Department of Health Sciences and Kinesiology, Crean College of Health and Behavioral Sciences, Chapman UniversityOrange, CA, USA; ^2^Department of Biological Sciences, Human and Evolutionary Biology Section, Dana and David Dornsife College of Letters, Arts and Sciences, University of Southern CaliforniaLos Angeles, CA, USA

**Keywords:** myokines, skeletal muscle glucose uptake, Jak/STAT, AMPK, IL-15

## Abstract

Myokines are specialized cytokines that are secreted from skeletal muscle (SKM) in response to metabolic stimuli, such as exercise. Interleukin-15 (IL-15) is a myokine with potential to reduce obesity and increase lean mass through induction of metabolic processes. It has been previously shown that IL-15 acts to increase glucose uptake in SKM cells. However, the downstream signals orchestrating the link between IL-15 signaling and glucose uptake have not been fully explored. Here we employed the mouse SKM C2C12 cell line to examine potential downstream targets of IL-15-induced alterations in glucose uptake. Following differentiation, C2C12 cells were treated overnight with 100 ng/ml of IL-15. Activation of factors associated with glucose metabolism (Akt and AMPK) and known downstream targets of IL-15 (Jak1, Jak3, STAT3, and STAT5) were assessed with IL-15 stimulation. IL-15 stimulated glucose uptake and GLUT4 translocation to the plasma membrane. IL-15 treatment had no effect on phospho-Akt, phospho-Akt substrates, phospho-AMPK, phospho-Jak1, or phospho-STAT5. However, with IL-15, phospho-Jak3 and phospho-STAT3 levels were increased along with increased interaction of Jak3 and STAT3. Additionally, IL-15 induced a translocation of phospho-STAT3 from the cytoplasm to the nucleus. We have evidence that a mediator of glucose uptake, HIF1α, expression was dependent on IL-15 induced STAT3 activation. Finally, upon inhibition of STAT3 the positive effects of IL-15 on glucose uptake and GLUT4 translocation were abolished. Taken together, we provide evidence for a novel signaling pathway for IL-15 acting through Jak3/STAT3 to regulate glucose metabolism.

## Introduction

Obesity is a major problem in our modern society, its prevalence continues to grow, and it is linked to many disease processes, such as diabetes, cardiovascular disease, and certain cancers (Félix-Redondo et al., 2013; Ogden et al., 2014). A multitude of studies have been underway to uncover regulators of metabolism with the intent of treating and/or preventing obesity and its associated disease states (Hampton, 2012; Pedersen and Febbraio, 2012; Egan and Zierath, 2013; Febbraio, 2014). It has long been known that increasing skeletal muscle (SKM) mass and activity are routes to induce energy expenditure for the reduction of adiposity and improvements in insulin resistance (Ivy et al., 1986; Kraegen et al., 1989; Pedersen and Febbraio, 2012). Recently, SKM has proven to possess functions beyond muscle contraction (Pedersen and Febbraio, 2012). Many cytokines secreted from skeletal muscle, termed “myokines,” have been identified and muscle contraction appears to be a major stimulator of their release (Pedersen, 2013; Raschke and Eckel, 2013; Catoire et al., 2014; Eckardt et al., 2014). Multiple myokines, BDNF5, FGF21, irisin, and interleukin-6 (IL-6), among others, have been shown to exert their positive metabolic actions in an endocrine/paracrine manner (Fisher ffolliott et al., 2012; Raschke and Eckel, 2013; Catoire et al., 2014; Indrakusuma et al., 2015).

Interleukin-15 (IL-15) is a myokine that has shown promise for the prevention and/or treatment of obesity and metabolic disorders (Alvarez et al., 2002; Argilés et al., 2009; Quinn and Anderson, 2011; Sun and Liu, 2015). Historically, IL-15 has been regarded as an activator of natural killer (NK) cells and has been viewed as pro- and anti-inflammatory with anti-tumorigenic potential (Castillo and Schluns, 2012; Lutz and Quinn, 2012). IL-15 belongs to a 4-α-helix bundle cytokine family alongside a variety of other interleukins that are within this family (Budagian et al., 2006; Waldmann, 2015). Expression of the gene encoding for IL-15 results in the translation of two isoforms, a short 21-amino acid and a long 48-amino acid signal peptide (Quinn and Anderson, 2011). The long IL-15 isoform combines intracellularly with its receptor, IL-15Rα, prior to secretion from SKM (Budagian et al., 2006; Quinn and Anderson, 2011). Subsequently, the cytokine-receptor complex either trans-presents or binds to the β and γ chains of the IL-2 receptor on its target tissues for initiation of IL-15 signaling (Budagian et al., 2006; Castillo and Schluns, 2012). Alternatively, IL-15 can form a heterodimer with the IL-2 receptor independent of IL-15Rα (Castillo and Schluns, 2012). Importantly, there is evidence that the appearance of IL-15 in circulation increases following exercise, in humans and rodents, although this notion is somewhat controversial (Gray and Kamolrat, 2011; Catoire et al., 2014; Rinnov et al., 2014; Crane et al., 2015; Pierce et al., 2015).

Increased circulating levels of IL-15 have been implicated in stimulating expression of mitochondrial associated factors, such as PPARs and SIRT1, in mouse SKM (Almendro et al., 2008; Quinn et al., 2012, 2013; O'Connell and Pistilli, 2015). Additionally, IL-15 has the ability to stimulate both glucose uptake and fatty acid oxidation in SKM cells (Busquets et al., 2006). Further, transgenic mice with increased IL-15 in circulation display an increased endurance capacity phenotype (Quinn et al., 2012). However, recent *in vivo* studies have questioned the relevance of IL-15 secretion following exercise in humans (Pierce et al., 2015). Although it has been demonstrated that IL-15 induces metabolic pathways in SKM, the discrete molecular mediators of these effects have not been fully defined. The most well studied pathway for IL-15 action is the janus kinase activation of signal transducer and activator of transcription proteins (Jak/STAT) signaling pathway (Waldmann, 2015; Ye, 2015). Upon IL-15 binding to the IL-2 receptor, Jak isoforms (Jak1 and/or Jak3) are auto-phosphorylated and in turn induce phosphorylation of STAT3 and/or STAT5 (Ye, 2015). Overall the Jak/STAT signaling pathway has a large number of intracellular functions with the potential to effect energy metabolism in many cell types (Frias and Montessuit, 2013; Richard and Stephens, 2014; Ye, 2015). Alternatively, pathways aside from the Jak/STAT signaling cascade have been linked to IL-15 action (Stone et al., 2011; Zhao and Huang, 2012; Crane et al., 2015; Waldmann, 2015). For instance, it has been established that the PI3K/Akt pathway becomes activated downstream of IL-15 action (Budagian et al., 2006; Zhao and Huang, 2012; Lai et al., 2013; Waldmann, 2015; Ye, 2015). Additionally, a link between the energy sensing enzyme AMP-activated protein kinase (AMPK) and IL-15 has been established by us and others (Abbott et al., 2012; Turcotte and Abbott, 2012; Crane et al., 2015). Both Akt and AMPK signaling exert beneficial effects on substrate metabolism such as glucose uptake and fatty acid oxidation in SKM cells, in line with IL-15 action (Thorell et al., 1999). However, little is known regarding the signaling pathway downstream of IL-15-IL-2 receptor interaction, to mediate substrate metabolism in SKM cells.

Overall, there is strong evidence that IL-15 plays a positive role in mediating SKM substrate utilization (Busquets et al., 2006; Argilés et al., 2009; Quinn and Anderson, 2011). However, the signaling molecules responsible for orchestrating IL-15 action on energy metabolism have yet to be firmly established in SKM. The purpose of this study was to identify the molecular pathways that mediate the downstream effects of IL-15 signaling in SKM cells. Here, we demonstrate that IL-15 increases glucose uptake and GLUT4 translocation, through induction of the Jak3/STAT3 signaling pathway in SKM cells.

## Methods

### C2C12 cell culture

The immortalized mouse SKM fibroblast line, C2C12 (Sigma), was cultured in DMEM supplemented with 10% fetal bovine serum (FBS; Sigma), 1% Penicillin-Streptomycin (10,000 U/mL; Corning), and 0.1% Amphotericin B (Corning). At 80% confluence, cells were induced toward differentiation to mature myotubes with DMEM supplemented with 2% horse serum (Sigma) and 1 μM insulin (Sigma) for 6 days. Differentiation was confirmed by visualization of myotube formation. On the fifth day of differentiation cells were treated with 100 ng/ml of recombinant IL-15 (Genscript) for 24 h, as previously described (Abbott and Turcotte, 2014; Thornton et al., 2016).

### Glucose uptake assay

Glucose uptake was measured in fully differentiated C2C12 cells using a non-radioactive fluorometric assay, as previously described (Leira et al., 2002; Zou et al., 2005; Kanwal et al., 2012). Briefly, following differentiation in 6 well plates, cells were serum starved in growth media for 2 h and cells were treated with either vehicle control, IL-15 100 ng/ml, insulin (100 nM), a STAT3 inhibitor (100 μM; S31-201; Sigma), or IL-15 + S31-20 with or without 50 μM 2-(N-(7-Nitrobenz-2-oxa-1,3-diazol-4-yl)Amino)-2-Deoxyglucose (2-NBDG) (Cayman Chemical) in Krebs-Henseleit buffer containing 0.1% fatty acid free BSA (Sigma) and 2000 mg/L of glucose at 37°C with 5% CO_2_(Siddiquee et al., 2007; Kelly et al., 2010). Following 2 h, the cells were lysed in PBS-Triton-X100 buffer and florescence was measured using a microplate reader with excitation 488 nm and emission 520 nm. The concentration of NBDG uptake was calculated from a standard curve of NBDG in lysis buffer. Values were normalized to the protein content present in the cell lysates.

### Western blot analysis

C2C12 cells were lysed in a modified RIPA buffer supplemented with protease inhibitors (Pierce) (Abbott et al., 2009). For nuclear fractionation, cells were lysed with a commercially available kit and cytosolic and nuclear fractions were recovered (Pierce) (Abbott et al., 2012). Total protein was assessed using a standard Bradford assay (BioRad). Approximately 20 μg of protein from the cell homogenate preparations were separated on a 4–12% gradient gel (GenScript) via SDS-PAGE. Proteins were transferred onto Immobilon-P polyvinylidene difluoride (PVDF) membranes and blocked with 5% BSA in Tween-TBS for 1 h The membranes were then incubated (4°C) in 5% BSA in Tween-TBS with antibodies (1:1000) against phospho-AMPK-Thr172, total AMPK, phospho-Akt-Thr308, phospho-Akt substrate, total Akt, phospho-Jak1, total Jak1, phospho-Jak3, total Jak3, phospho-STAT3-Tyr705, total STAT3, phospho-STAT5, total STAT5 (Cell Signaling), GLUT4 (Santa Cruz), and GAPDH (Sigma). Following overnight incubation, the membranes were then probed with a secondary antibody (Genscript, 1:2000; or Thermo, 1:10,000). Blots were then washed and subjected to enhanced chemiluminescence (Pierce). Band density was quantified using Image J and normalized to control samples. Membranes were stripped in 0.5 M NaOH for 15 min at room temperature, followed by three 5 min washes in TBS-T, and subsequently probed for total proteins and subsequently GAPDH (Sigma) was used as a loading control.

### Immunofluorescence

After overnight IL-15 experimental treatment, the C2C12 cells were fixed with ice cold methanol. Cells were blocked with 5% BSA in PBS-Tween followed by a series of washes. Cells were incubated overnight in a humidified chamber with primary antibodies (GLUT4 or pSTAT3) at 4°C. Alexaflour-488 (Molecular Probes) secondary antibodies were used and DAPI was used as a nuclear stain. Cells were imaged using a Zeiss inverted microscope and images were captured with an Axiovision camera. Quantification of GLUT4 on the plasma membrane and co-localization of STAT3 to the nuclei was performed using Image J and Fiji software (Schindelin et al., 2012; Schneider et al., 2012).

### Immunoprecipitation

After the experimental treatments, cells were lysed as described in Western blot analysis and Jak3 specific antibodies (Cell Signaling) were added to the lysate (250 ug) and incubated overnight at 4°C, as previously described (Abbott et al., 2009; Bogachus and Turcotte, 2010; Ahmadian et al., 2011). Protein A/G agarose beads were added and the slurry was incubated for 2–4 h at 4°C (Santa Cruz Biotechnology). The immunoprecipitates were collected by centrifugation and pellets were washed with PBS buffer, and the final supernatants were resuspended in SDS-sample buffer (Genscript). Subsequently western blotting procedures were carried out as previously described and antibodies specific to STAT3 (Cell Signaling) were used. Ponceau S was applied to the membranes to verify equal protein loading.

### Real time PCR

Standard RNA isolation procedures were performed on the cells following the 6 day treatment protocol, as previously described (Pedersen and Febbraio, 2012; Ogden et al., 2014). Briefly, cells were lysed with Trizol reagent and chloroform was added to separate the RNA from the DNA and protein fractions. RNA was precipitated from the clear phase of the Trizol-chloroform mixture, followed by centrifugation at 12,000 g at 4°C, with isopropanol. The RNA pellet was washed with 75% ethanol, centrifuged at 7500 g for 5 min, at 4°C, and the pellets were air-dried. Using RNAse-free water, the pellets were resuspended and the RNA purity and concentration were quantified using a Nano-Drop spectrophotometer. Reverse transcription of RNA to cDNA was performed on 2 ug of RNA using SuperScript reverse transcriptase VILO kit. Real time qPCR was performed on the cDNA, using SYBR-green in a 96 well plate. Primer sequences for HIF1α, SOCS3, and GAPDH are listed in Table 1. GAPDH was used as an internal control and the ddCT method was used to calculate gene expression levels.

**Table 1:** **Primer sequences used for qPCR analysis**.

**Gene**	**Sequence**
HIF1α—F	GCACTAGACAAAGTTCACCTGAGA
HIF1α—R	CGCTATCCACATCAAAGCAA
SOCS3—F	ATTTCGCTTCGGGACTAGC
SOCS3—R	AACTTGCTGTGGGTGACCAT
Gapdh—F	AGGTCGGTGTGAACGGATTTG
Gapdh—R	TGTAGACCATGTAGTTGAGGTCA

### Statistics

Statistical analysis was carried out using GraphPad Prism 6.0 software. Values are displayed as means ± SEM. Each experiment consisted of 3 internal replicates and then were repeated 2–3 times for a total *n* = 6–9 for each experimental group. IL-15 treatment values were compared to vehicle control cells and a Student's *T*-test was calculated with *P* < 0.05 considered to be statistically significant. For glucose uptake assessments a One-way ANOVA was used with a Fisher's post hoc analysis and a *P*-value of <0.05 was considered to be statistically significant.

## Results

### IL-15 promotes GLUT4 translocation and glucose uptake

**Figure 1:**
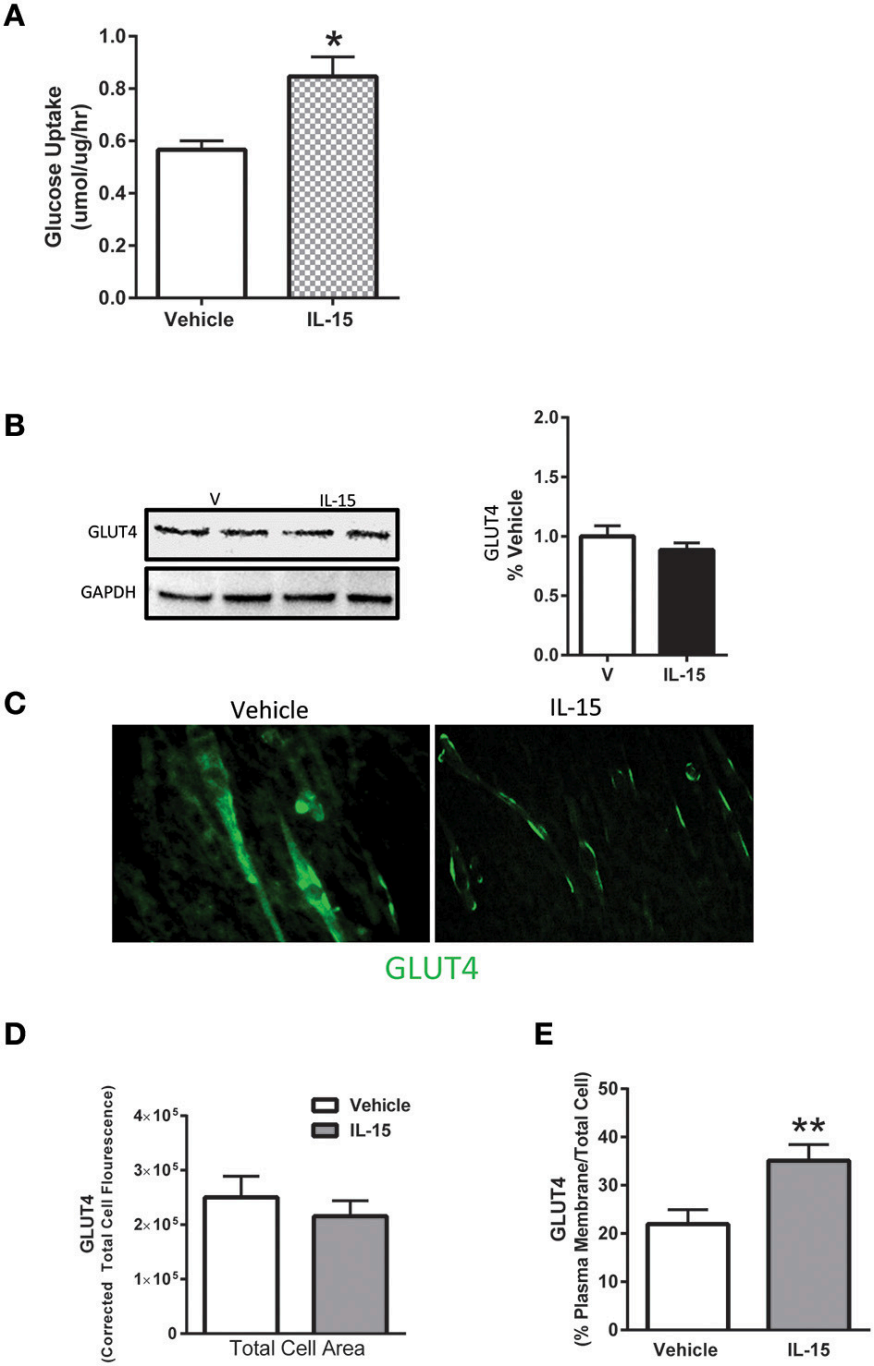
**Effect of IL-15 signaling on glucose uptake and GLUT4. (A)** Glucose uptake with IL-15 using a 2-NBDG non-radioactive assay; **(B)** western blotting of GLUT4 protein expression in whole cell lysates; **(C)** immunofluorescence of GLUT4 in the fully differentiated myotubes; **(D)** quantification of GLUT4 expression as indicated by fluorescence within the total cell area; **(E)** percent of GLUT4 expression in the plasma membrane relative to the total GLUT4 within the cell. Assessments were carried out on differentiated C2C12 myotubes following 24 h treatment with 100 ng/ml of IL-15 for the western blotting. All values are displayed as means ± SEM, *n* = 6 per group, ^*^P < 0.05, ^**^P < 0.005.

In line with previously reported data (Busquets et al., 2006), IL-15 treatment resulted in an increase in glucose uptake by 49% when compared to the control SKM cells (*P* < 0.05; Figure 1A). However, unlike mRNA expression data reported by others (Busquets et al., 2006), there was no significant effect of IL-15 on the total cell protein content of GLUT4 (*P* > 0.05; Figure 1B). Conversely, IL-15 treatment stimulated a translocation of GLUT4 from the intracellular compartment to the plasma membrane of the fully differentiated SKM myotubes (Figure 1C). Similar to the western blot expression there were no differences in the quantifiable immunofluorescence of GLUT4 in total cell area (*P* > 0.05; Figure 1D). However, the percentage of GLUT4 present on the plasma membrane relative to the total GLUT4 expression was significantly greater in the IL-15 treated cells (*P* < 0.05; Figure 1E).

### IL-15 mediated effects on enzyme activation and protein expression of IL-2R targets

**Figure 2:**
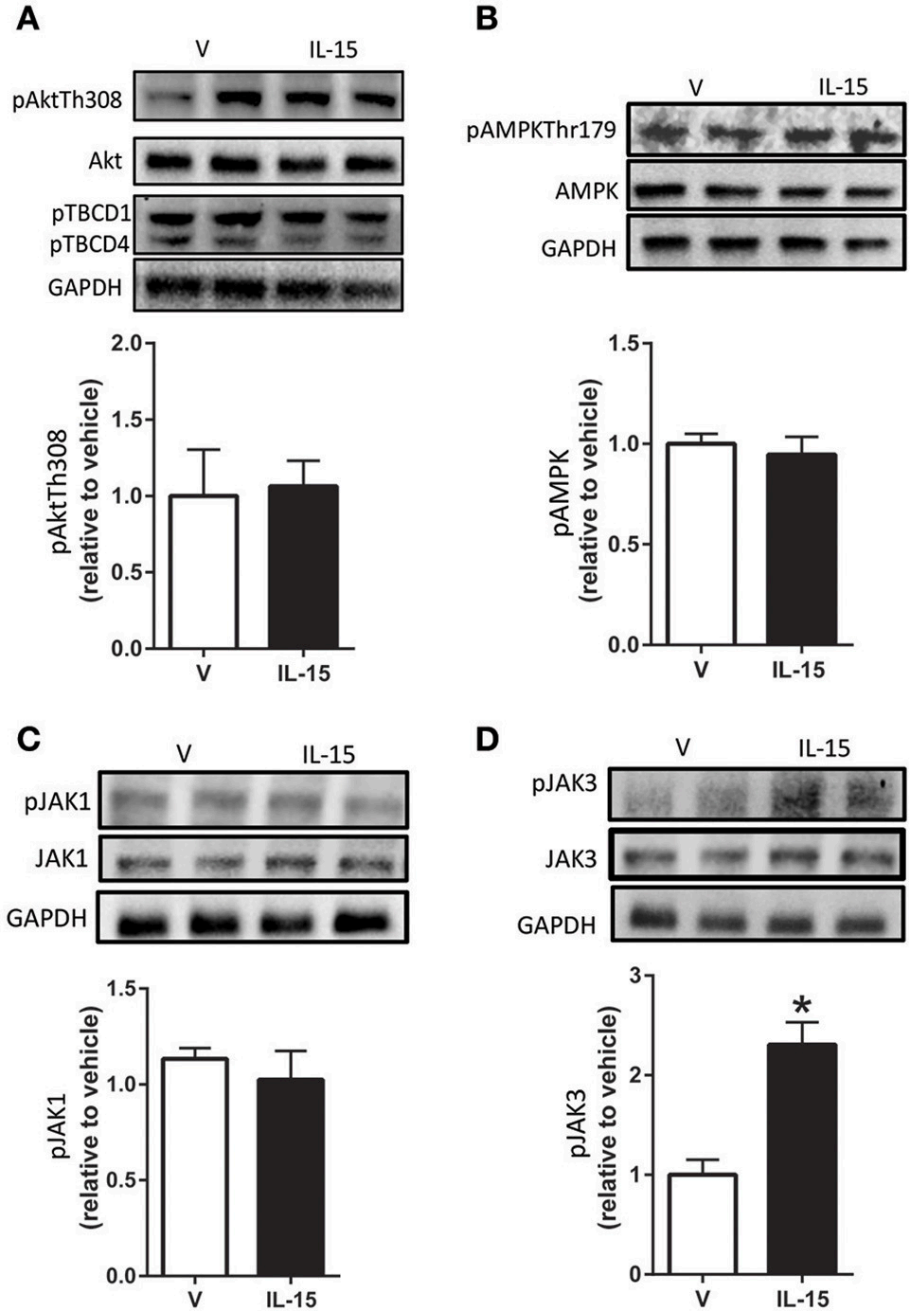
**Effects of IL-15 signaling on mediators of glucose uptake and proteins downstream of IL-2βγ receptor signaling**. Western blotting of **(A)** phospho-Akt-Thr308, TBCD1, TBCD4, and total Akt; **(B)** phospho-AMPK-Thr172 and total AMPK; **(C)** phospho-Jak1 and total Jak1; **(D)** phospho-Jak3 and total Jak3. Assessments were carried out on whole cell lysates from differentiated C2C12 myotubes following 24 h treatment with 100 ng/ml of IL-15. GAPDH was used as a loading control for western blotting. Vehicle cells were used as a control to calculate protein expression. Image J was used to quantify band density in western blotting experiments. All values are displayed as means ± SEM, *n* = 6–9 per group, ^*^P < 0.05.

Despite previous evidence that IL-15 may signal through Akt in lymphocytes, we were unable to detect any alterations in phospho-Akt or total Akt protein expression (*P* > 0.05; Figure 2A, (Lai et al., 2013; Waldmann, 2015)). Further, the phosphorylation state of downstream substrates (TBC1D1 and TBC1D4) of Akt were also not altered with IL-15 (*P* > 0.05; Figure 2A). AMPK is a master metabolic regulator and is confirmed to stimulate GLUT4 translocation along with glucose uptake (Winder and Hardie, 1999; Abbott et al., 2009). Additionally, a link between AMPK and IL-15 has been identified by us and others (Abbott et al., 2012; Crane et al., 2015). However, there were no alterations in phosphorylated AMPK at Thr179 or total protein expression of AMPK with IL-15 treatment (*P* > 0.05; Figure 2B).

Because IL-15 shares a receptor with IL-2, we attempted to examine potential signaling molecules directly downstream of the IL-2 receptor in SKM cells (Budagian et al., 2006; Waldmann, 2015). A widely studied route of IL-15 signaling is through activation of the Jak/STAT pathway (Mishra et al., 2014b; Waldmann, 2015). However, IL-15 treatment failed to induce an increase in phosphorylation of Jak1 at Tyr1022/1023 (*P* > 0.05; Figure 2C). Likewise, there were no effects of IL-15 treatment in regards to total protein expression of Jak1 (Figure 2C). Conversely, in the presence of IL-15, phosphorylation of Jak3 at Tyr980/981 was increased (130%) with no alterations in its total protein expression (*P* < 0.05; Figure 2D).

**Figure 3:**
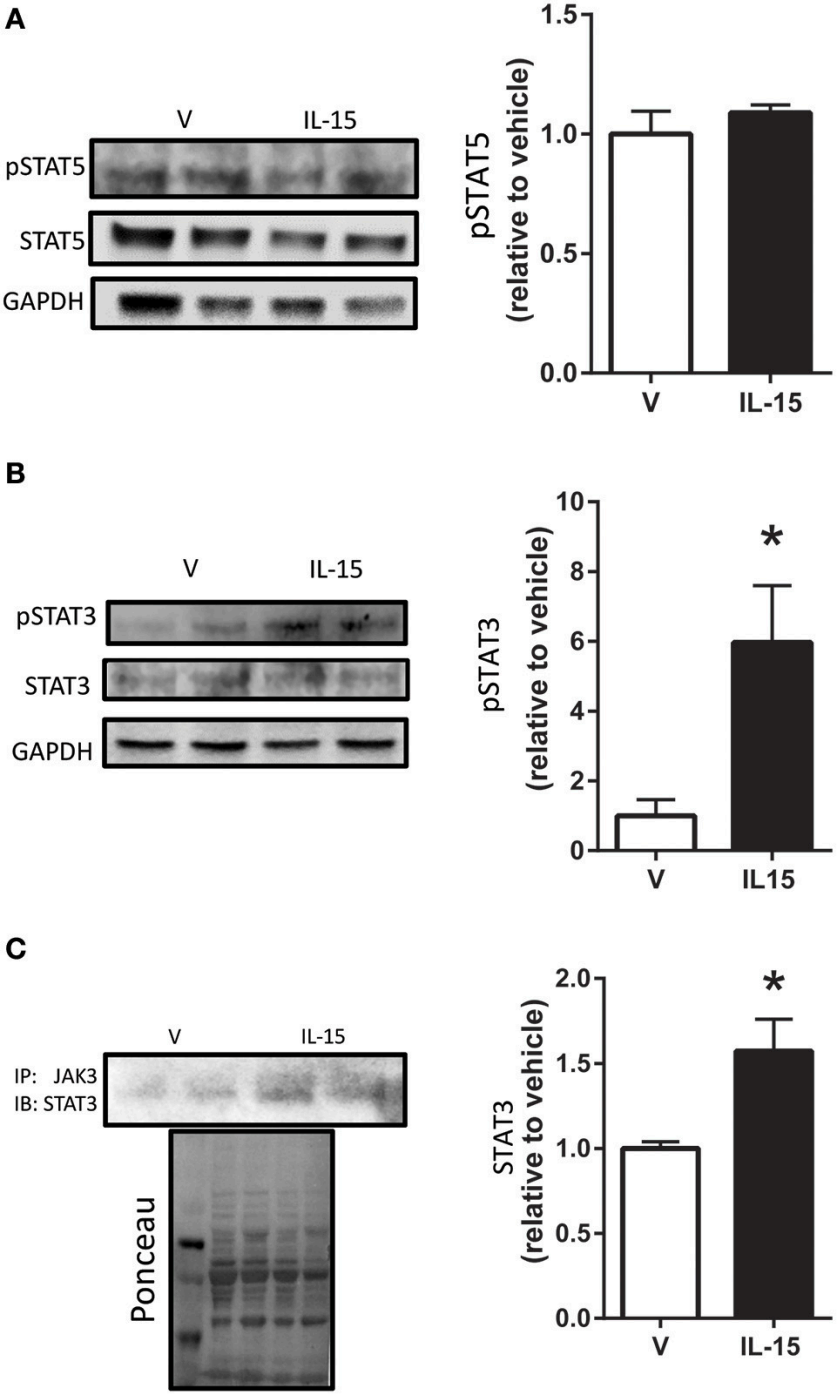
**Effects of IL-15 on STAT activation**. Western blotting of **(A)** phospho-STAT5 and total STAT5; **(B)** phospho-STAT3 at Tyr705 and total STAT3; **(C)** immunoprecipitation of Jak3 and subsequent western blotting of STAT3. Assessments were carried out on differentiated C2C12 myotubes following 24 h treatment with 100 ng/ml of IL-15. GAPDH was used as loading control for western blotting. Ponceau S was used as a loading control for the immunoprecipitation analysis. Vehicle cells were used as a control to calculate protein expression. Image J was used to quantify band density in western blotting experiments. All values are displayed as means ± SEM, *n* = 6 per group, ^*^P < 0.05.

**Figure 4:**
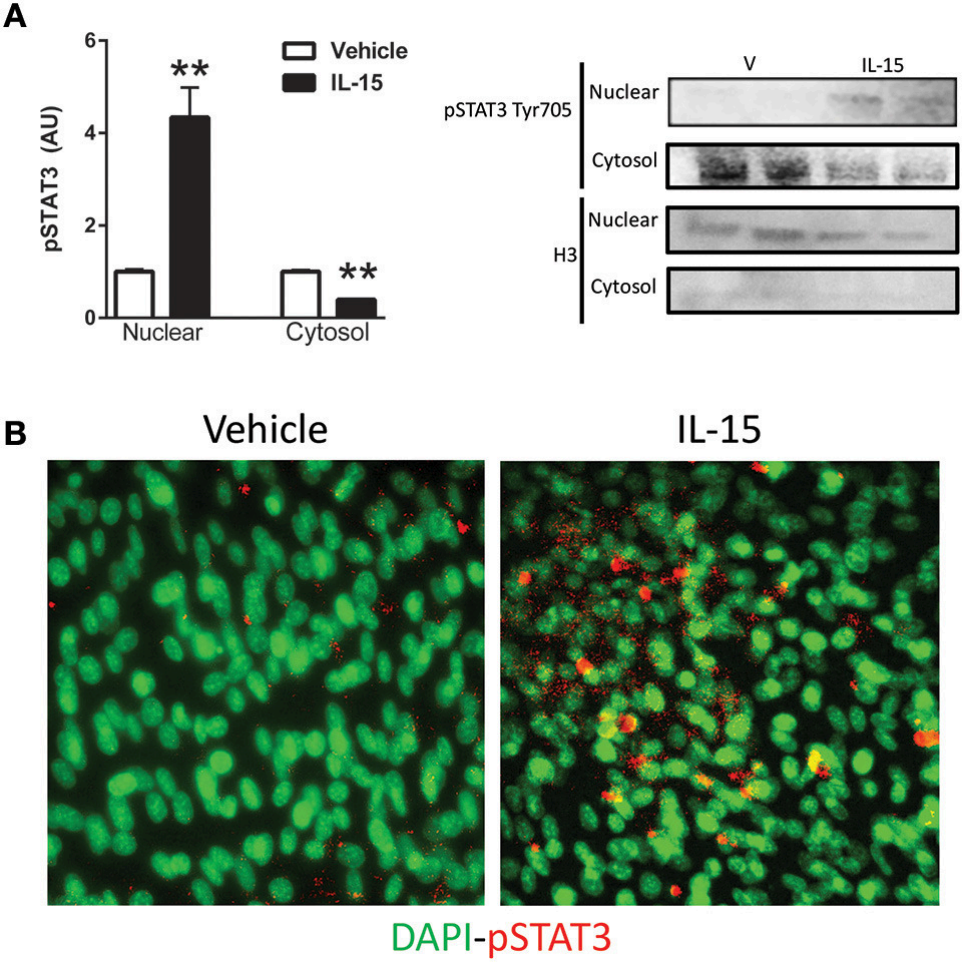
**IL-15 induced activation and translocation of STAT3. (A)** Phospho-STAT3 at Tyr705 in the nuclear and cytosolic fractions; and **(B)** representative image of phospho-STAT3 co-localization with nuclei by immunoflouresence. Assessments were carried out on differentiated C2C12 myotubes following 24 h treatment with 100 ng/ml of IL-15. Nuclear specific Histone 3 (H3) was used as verification of purity of nuclear fractions. Vehicle cells were used as a control to calculate protein expression. Image J was used to quantify band density in western blotting experiments. All values are displayed as means ± SEM, *n* = 6 per group, ^**^P < 0.05.

STAT5 is a known down-stream effector of Jak3 signaling, but phosphorylation of STAT5 at Tyr694 remained unchanged with the IL-15 treatment protocol (*P* > 0.05; Figure 3A). A less studied target of Jak3 is STAT3 and we measured increased phosphorylation of STAT3 at Tyr705, nearly 4-fold, with IL-15 (*P* < 0.05; Figure 3B). We also measured increased (57%) interaction between Jak3 and STAT3 in the presence of IL-15 (*P* < 0.05; Figure 3C). STAT3 has been shown to translocate to the nucleus, upon phosphorylation at Tyr705, to carry out its transcriptional activities (Tammineni et al., 2013). Here we measured a 3-fold increase in phosphorylated (Tyr705) STAT3 in the nuclear fraction as assessed by western blotting and immunofluorescence (*P* < 0.05; Figures 4A,B). The increases in phospho-STAT3 on the nuclear fraction corresponded to a 62% reduction of phospho-STAT3 in the cytosolic fraction (*P* < 0.05; Figure 4A).

### Involvement of STAT3 in mediating IL-15 induced glucose uptake

**Figure 5:**
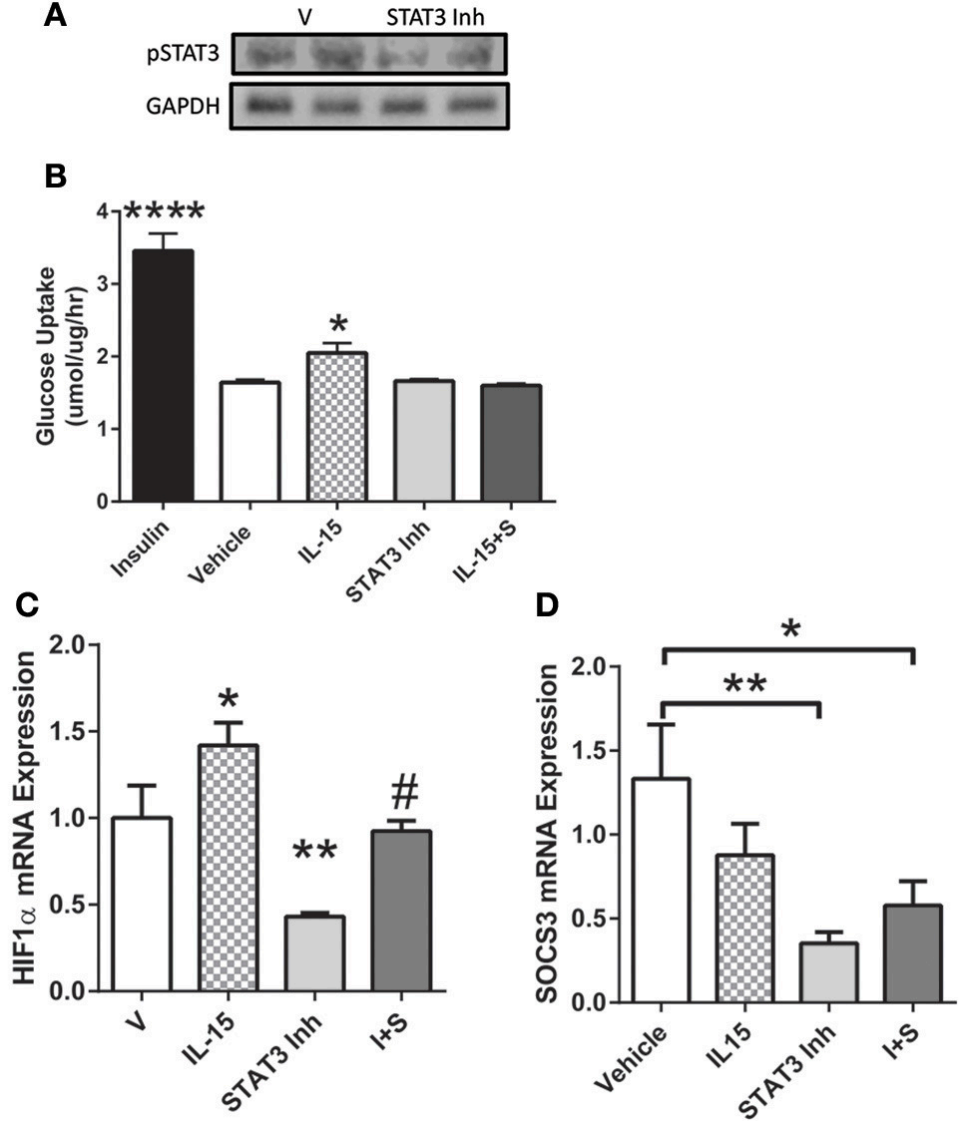
**Importance of STAT3 in IL-15 induced glucose uptake. (A)** inhibition of STAT3 was verified by western blotting against phospho-STAT3; **(B)** glucose uptake was assessed in the presence of vehicle control, insulin (100 nM), IL-15 (100 ng/ml), a STAT3 inhibitor (100 μM; S31-201), or with both IL-15 and the STAT3 inhibitor (IL-15 + S); **(C)** HIF1α mRNA expression levels; **(D)** SOCS3 mRNA expression levels. All values are displayed as means ± SEM, *n* = 6 per group, ^*^P < 0.05 (different from all groups); ^**^P < 0.005 (different from all groups); ^****^P < 0.0001 (different from all groups); #*P* < 0.05 (different from IL-15 group).

**Figure 6:**
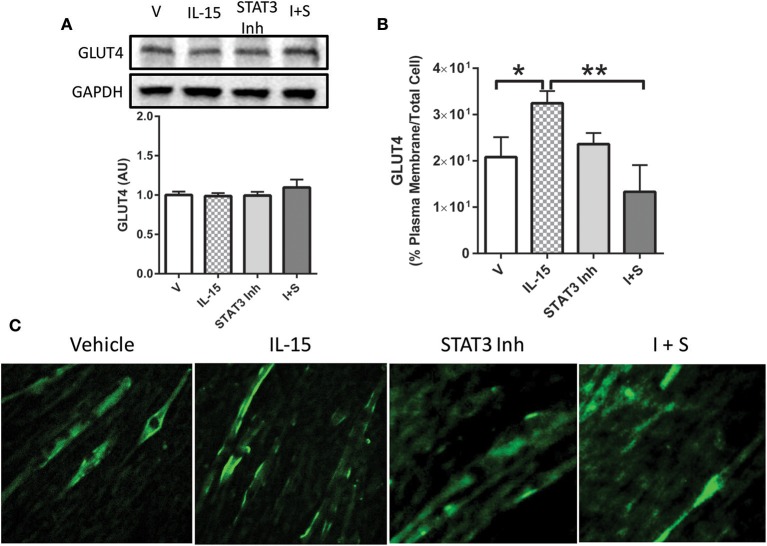
**Importance of STAT3 in IL-15 mediated GLUT4 translocation. (A)** GLUT4 expression; **(B)** percent of GLUT4 expression in the plasma membrane relative to the total GLUT4 within the cell; **(C)** immunofluorescence of GLUT4 in myotubes. Cells with treated with either vehicle control, IL-15 (100 ng/ml), a STAT3 inhibitor (100 μM; S31-201), or with both IL-15 and the STAT3 inhibitor (IL-15 + S). All values are displayed as means ± SEM, *n* = 6 per group, ^*^P < 0.05; ^**^P < 0.005.

In order to determine the necessity of STAT3 activation for mediating IL-15 induced glucose uptake, we used a STAT3 inhibitor. Inhibition of STAT3 in the SKM cells was verified by western blotting of pSTAT3 (*P* < 0.05; Figure 5A). When STAT3 was inactivated, the IL-15 induced increases in glucose uptake were abolished when compared to vehicle control cells (*P* < 0.05; Figure 5B). It should be noted that addition of the vehicle control (DMSO) in the inhibitor studies yielded alterations in the relative amounts of glucose uptake when compared to non-DMSO treated cells (Figure 1A). STAT3 has previously been shown to mediate glucose up through HIF1α induced translocation of GLUT4 (Demaria et al., 2010; Sakagami et al., 2014). Indeed IL-15 induced an increase in mRNA expression levels of HIF1α and these effects were prevented with STAT3 inhibition in the presence of IL-15 (*P* < 0.05; Figure 5C). Conversely, IL-15 had no effect on SOCS3 mRNA expression levels, while as expected, inhibition of STAT3 yielded reductions in SOCS3 expression levels (Figure 5D). Inhibition of STAT3 had no effects on total cellular GLUT4 expression (*P* > 0.05; Figure 6A). However, the IL-15 induced translocation of GLUT4 to the plasma membrane was abolished with STAT3 inhibition in combination with IL-15, as measured by percentage of GLUT4 on the plasma membrane relative to the total cell GLUT4 expression (*P* < 0.05; Figures 6B,C).

## Discussion

Data from this study solidifies the notion that the myokine IL-15 is directly involved in mediating glucose metabolism in SKM. Here we have brought to light a comprehensive signaling mechanism for IL-15 action in SKM cells. Our data shows that IL-15 promotes translocation of GLUT4 to the plasma membrane, accounting for the increase in glucose uptake. Additionally, our data indicates that IL-15 signals through the Jak3/STAT3 signaling pathway, without activation of other established downstream signals of IL-2R, in SKM cells. Moreover, we show for the first time that IL-15 promotes the translocation of phosphorylated STAT3 to the nucleus. The IL-15 induced translocation of STAT3 might provide insight into the molecular mechanism behind the stimulatory effect of IL-15 on overall cellular metabolism. Importantly, we uncovered a role for IL-15 induced STAT3 activation to induce HIF1α expression, leading to an increase in GLUT4 translocation to the plasma membrane. Altogether, we provide evidence that IL-15 signaling in SKM is tightly regulated through coordination of the Jak3/STAT3 signaling pathway to promote GLUT4 translocation and consequently glucose uptake.

IL-15 has been extensively studied in tissues and cells other than SKM cells, such as NK lymphocytes (Lai et al., 2013; Mishra et al., 2014a). IL-15 signaling is complex, in that, it is ubiquitously expressed, but its secretion is proposed to be limited to SKM, and it is secreted with its receptor, IL-15Rα (Budagian et al., 2006; Catoire et al., 2014). Complicating IL-15 signaling is its ability to trans-present in combination with IL-15Rα to neighboring cells (Castillo and Schluns, 2012; Waldmann, 2015). Nevertheless, once released into circulation the IL-15/IL-15Rα complex binds, or IL-15 acting alone, to the IL-2Rβ/γ chains on its target tissue (Giri et al., 1995). It has repeatedly been shown that IL-15 induces mitochondrial associated factors in various tissues, such as adipose and SKM (Almendro et al., 2006; Quinn et al., 2012, 2013; Barra et al., 2014; O'Connell and Pistilli, 2015). With this in mind, we examined the role of IL-15 signaling in mediating increases in substrate availability through modulation of transport proteins. We did not detect any alterations in total GLUT4 protein expression, which has been hypothesized to be controlled by IL-15 signaling (Busquets et al., 2006). However, in line with other studies, we measured increases in glucose uptake with IL-15 treatment in the SKM cells (Busquets et al., 2006). Further, we show that IL-15 acts to increase glucose uptake through induction of translocation of GLUT4 to the plasma membrane. GLUT4 translocation is a well-studied pathway and has been shown to occur via multiple regulators, such as AMPK (Thorell et al., 1999; Viollet et al., 2003). Additionally, it has been recognized that IL-15 acts upstream of proteins associated with mitochondrial activity, such as PGC1α, PPARs, and SIRT1 (Quinn et al., 2008, 2012; O'Connell and Pistilli, 2015). PGC1α and PPAR signaling are downstream targets of the important energy sensing enzyme, AMPK, and us and others, have shown a relationship between AMPK and IL-15 in SKM. We previously showed that treatment with IL-15 reduced phosphorylated AMPK content in SKM cells. However, in those experiments cells were serum starved, which yields independent effects on AMPK. In this regard, we maintained our cells, in the current studies, in growth media including serum, to rule out signaling pathways beyond IL-15 signaling. Nevertheless, we hypothesized that the beneficial metabolic effects of IL-15 may signal through activation of AMPK. However, upon IL-15 treatment, phosphorylation of AMPK remained unchanged (Abbott et al., 2012; Wan et al., 2014; Crane et al., 2015).

Binding of the IL-15/IL15Rα complex to IL-2Rβ/γ activates numerous signaling pathways, such as the Akt and Jak/STAT pathways, among others (Stone et al., 2011; Zhao and Huang, 2012; Lai et al., 2013). We speculated that IL-15 signals through the Akt signaling pathway, based on previously published work and our glucose kinetics data (Zhao and Huang, 2012). Interestingly, we were unable to measure increases in Akt activation by examination of its phosphorylation state and by assessing its downstream substrates, TBC1D1 and TBC1D4. The results of our investigation of Akt are in contradiction to other studies that point to a link between IL-15 and Akt signaling in SKM (Zhao and Huang, 2012; Lai et al., 2013). The study conducted by Zhao and Huang showed that IL-15 treatment, in combination with Akt overexpression, acted to regulate T-cell-SKM interaction following denervation (Zhao and Huang, 2012). However, in our study we examined the ability of IL-15 treatment to modulate the activation state of Akt in C2C12 SKM cells.

Ruling out AMPK and Akt activation, we next examined the Jak/STAT signaling pathway, which is widely accepted as a downstream modulator of IL-15/IL-2R signaling (Budagian et al., 2006; Lai et al., 2013; Waldmann, 2015; Ye, 2015). Not surprisingly, Jak3 phosphorylation was increased, as Jak3 has repeatedly been shown to be activated by IL-15/IL-2R signaling in numerous cells (Lai et al., 2013). On the other hand, some have shown that Jak1 is downstream of IL-15 signaling, but here, we failed to provide a link between IL-15 and Jak1 signaling, which is line with other reports (Kirken et al., 1995; Lai et al., 2013). Further, IL-15 failed to induce STAT5 phosphorylation, which is not in agreement with previous studies, showing STAT5 as downstream of the IL-2R and/or IL-15 signaling (Lin and Leonard, 2000; Crane et al., 2015). Of note, little is known about the signaling molecules downstream of the IL-15 receptor, IL-2R, in SKM cells, which may account for the discrepancies between our data an others published in alternate cell types. Nevertheless, our data indicates that IL-15 signaling induces activation of STAT3, which is in line with other studies in endothelial cells (Stone et al., 2011). Importantly, we measured increased Jak3-STAT3 interaction, presumably to induce STAT3 phosphorylation at Tyr705, in the IL-15 treated SKM cells. It has been previously determined that upon phosphorylation and activation, from an upstream Jak, STAT3 translocates to the nucleus to bind to DNA, inducing its transcriptional activity (Yahata et al., 2003; Frias and Montessuit, 2013). In line with these aforementioned reports, we measured increased phosphorylated STAT3 content in the nuclear fraction of the SKM cells with IL-15 induction. Thus, translocation of STAT3, following phosphorylation at Tyr705, to the nucleus is an indicator of its transcriptional activity and is the potential route by which IL-15 promotes expression of genes associated with metabolic activity (Bild et al., 2002; Yahata et al., 2003). Since STAT3 activation has been associated with increases in glucose metabolism, our data provides a link for IL-15 induced metabolic function through Jak3/STAT3 signaling (Cui et al., 2004; Frias and Montessuit, 2013; Richard and Stephens, 2014). On the other hand, it has been suggested that STAT3-SOCS3 signaling stimulates insulin resistance in high fat diet induced obesity (Wunderlich et al., 2013). However, we can rule out SOCS3 as a player in the IL-15 signaling pathway, in regards to glucose metabolism, as its expression levels remained unchanged in the presence of IL-15. Adding to the controversy, when STAT3 was knocked out of skeletal muscle, in mice, there were no effects on whole body insulin tolerance, glucose uptake, fat oxidation, physical activity, or body weight (White et al., 2015). Nevertheless, here we can conclude, based on our data, that STAT3 activation, via IL-15, is indeed involved in mediating glucose uptake in SKM myotubes. Here we show that HIF1α, an established downstream target of STAT3, and mediator of glucose uptake and GLUT4 translocation, may be the link between IL-15-Jak3/STAT3 and glucose uptake (Demaria et al., 2010; Sakagami et al., 2014).

Taken together, it is clear that downstream effects of the IL-15 signaling pathway, required to carry out its beneficial effects on metabolism, are complex and convoluted. Data presented here provide evidence for direct regulation of IL-15 on glucose uptake in SKM cells. Additionally, we solidify a signaling mechanism for IL-15 action, through activation of Jak3 to ultimately activate and promote STAT3 translocation to the nucleus. We propose a novel signaling mechanism for IL-15 mediated increases in glucose uptake and GLUT4 translocation via nuclear translocation of STAT3. Further studies are essential to firmly establish the unknown factors involved in the IL-15-Jak3/STAT3 signaling axis for the potential treatment and/or prevention of metabolic disorders, such as insulin resistance or diabetes.

## Author contributions

JK, ST, and MA performed experiments and data collection. JK and ST edited and approved manuscript. MA devised experiments, performed statistical assessments, and wrote manuscript.

## Funding

This work was supported by Chapman University, the American College of Sports Medicine Foundation, and the American Heart Association Grant No. 16SDG30680003 (MA).

### Conflict of interest statement

The authors declare that the research was conducted in the absence of any commercial or financial relationships that could be construed as a potential conflict of interest.
